# Microbial Community Structure and Functional Potential Along a Hypersaline Gradient

**DOI:** 10.3389/fmicb.2018.01492

**Published:** 2018-07-10

**Authors:** Jeffrey A. Kimbrel, Nicholas Ballor, Yu-Wei Wu, Maude M. David, Terry C. Hazen, Blake A. Simmons, Steven W. Singer, Janet K. Jansson

**Affiliations:** ^1^Microbial Communities Group, Deconstruction Division, Joint BioEnergy Institute, Emeryville, CA, United States; ^2^Physical Biosciences Division, Lawrence Berkeley National Laboratory, Berkeley, CA, United States; ^3^Biological and Systems Engineering Division, Lawrence Berkeley National Laboratory, Berkeley, CA, United States; ^4^Department of Microbiology, Oregon State University, Corvallis, OR, United States; ^5^Earth Sciences Division, Lawrence Berkeley National Laboratory, Berkeley, CA, United States; ^6^Earth and Biological Sciences Directorate, Pacific Northwest National Laboratory, Richland, WA, United States

**Keywords:** microbial communities, halophiles, biofuels, metagenomes, 16S rRNA

## Abstract

Salinity is one of the strongest environmental drivers of microbial evolution and community composition. Here we aimed to determine the impact of salt concentrations (2.5, 7.5, and 33.2%) on the microbial community structure of reclaimed saltern ponds near San Francisco, California, and to discover prospective enzymes with potential biotechnological applications. Community compositions were determined by 16S rRNA amplicon sequencing revealing both higher richness and evenness in the pond sediments compared to the water columns. Co-occurrence network analysis additionally uncovered the presence of microbial seed bank communities, potentially primed to respond to rapid changes in salinity. In addition, functional annotation of shotgun metagenomic DNA showed different capabilities if the microbial communities at different salinities for methanogenesis, amino acid metabolism, and carbohydrate-active enzymes. There was an overall shift with increasing salinity in the functional potential for starch degradation, and a decrease in degradation of cellulose and other oligosaccharides. Further, many carbohydrate-active enzymes identified have acidic isoelectric points that have potential biotechnological applications, including deconstruction of biofuel feedstocks under high ionic conditions. Metagenome-assembled genomes (MAGs) of individual halotolerant and halophilic microbes were binned revealing a variety of carbohydrate-degrading potential of individual pond inhabitants.

## Introduction

Microbes living in high-salt environments have developed strategies to survive the stresses of extremely saline conditions, particularly osmotic stress, and the need to retain turgor pressure for proper membrane bioenergetics (Oren, 1999). Salt stress is perhaps the most important environmental factor that influences bacterial community composition (Lozupone and Knight, 2007). Many bacteria and archaea can withstand ranges of salinity and balance osmotic stresses by increasing intracellular concentrations of solutes such as salt ions for “salt-in” microbes, and osmolytes for “salt-out” organisms (Ventosa et al., 1998; Oren, 2002). Due to the energetic expense of creating osmolytes in extremely saline conditions (Oren, 1999), microbes utilizing the salt-in strategy largely dominate hypersaline environments. Salt-in microbes include the order Halanaerobiales, archaea from the Halobacteria class and the bacterium *Salinibacter ruber*, the latter two also possess an extremely acidic proteome (Oren, 2013). Because of these drastic alterations to the genome and proteome of some salt-in microbes, their dependence on high salt concentrations often limits their abundance in low-salt environments.

Here we sought to determine how salinity impacts the microbial community structure and functional potential by studying a range of salinities from 2.5 to 33.2%. We sampled decommissioned industrial saltern ponds undergoing restoration to natural waterfowl habitat at the Eden Landing Ecological Reserve and Alviso Ponds in the South Bay near San Francisco, CA (Athearn et al., 2012). Water and sediment samples were collected from selected ponds along the salinity gradient and the community compositions were determined by the sequencing of 16S rRNA genes. To gain insights into the range of salinities the different classifications of microbes can tolerate, network analysis was done to identify groups with similar abundances among the sampled ponds, indicating similar abilities to tolerate osmotic stress.

Additionally, we obtained metagenomes and metagenome-assembled genomes (MAGs) to predict the metabolic potential of the pond communities, including polysaccharide utilization. Because of their tolerance to high temperature and ion concentrations (Delgado-García et al., 2012), proteins from halophilic organisms are of interest as industrial enzymes, particularly for the liberation of fermentable sugars from lignocellulosic biomass for advanced biofuels. From an industrial-use point of view, enzymes from salt-in organisms are potentially better candidates for fermentation under high-saline conditions because the proteins are more adapted to high salt ions, compared to proteins from organisms using the osmolyte strategy. Further, there are reports of enzymes in which salt has a stabilizing or even enhancing effect on activity of purified enzymes (Hirasawa et al., 2006; Voget et al., 2006; Gao et al., 2010; Zhang et al., 2011).

## Materials and methods

### Sampling

Water and sediment samples were collected from December 1 through December 9, 2012, from Ponds 2C and 1C in the Eden Landing Ponds, and A23 from the Alviso Ponds in the South Bay of San Francisco, CA, representing salinities of 2.5, 7.5, and 33.2%, respectively (Table 1). Each pond was sampled in three random locations by collecting sediment cores and water filtrate samples for DNA extraction and chemical analysis. Water samples were obtained by filtering 1 L of surface water through a 0.22 μm Sterivex filter, collecting a total of 5 L per site (EMD Millipore, Billerica, MA), followed immediately by 10 ml of RNA Later (Life Technologies, NY). Filters saturated with RNA Later were immediately stored on dry ice until placed at −80°C 3–5 h later. Sediment cores with a diameter of 45 mm were taken using a sludge sampler and immediately placed on dry ice for transport to the lab, whereupon they were stored at −80°C. Samples from sediment cores were excised as previously described (Mason et al., 2014), however the perimeter of each disc was discarded with a sterile scalpel to remove contamination from the coring process. Samples were prepared for DNA isolation by lysozyme and proteinase K treatment, followed by extraction with CTAB/phenol/chloroform with isopropanol/ethanol precipitation (Deangelis et al., 2013).

**Table 1:** Chemical analysis of pond water and sediments.

**Pond averages**	**Pond 2C**	**Pond 1C**	**Pond A23**
Temperature	10.5°C	11.0°C	11.0°C
Salinity (% ± standard error of the mean)	2.47 ± 0.03	7.57 ± 0.03	33.20 ± 0.00
pH (mean ± standard error of the mean)	8.65 ± 0.01	8.23 ± 0.01	7.41 ± 0.04
Latitude/Longitude	N 37 34.15, W 122 6.114	N 37 34.145, W 122 6.196	N 37 28.523, W 121 58.374
Total Dissolved Solids [g/L]	42.9	85	423.3

*Average of three replicates for each sample site*.

### 16S rRNA gene and metagenome sequencing

16S rRNA genes (515F/806R) and total metagenomic DNA were sequenced using the Illumina MiSeq (1 × 250 bp) and HiSeq (2 × 101 bp) platforms, respectively, at the Joint Genome Institute (JGI). Operational Taxonomic Units (OTU) at >97% sequence similarity were generated with the JGI iTagger pipeline (Tremblay et al., 2015). OTUs with < 10 total reads across all 18 samples were removed. Taxonomy of OTUs was assigned using the Ribosome Database Project (RDP) Classifier Version 2.11 (Wang et al., 2007). Chloroplast sequences were identified by searching against the ChloroplastDB reference database (Cui et al., 2006). Further characterization of individual OTUs to identify the closest sequenced genome was done by MegaBLAST search against the NCBI refseq_genomic database (Camacho et al., 2009). Principal Component Analysis and Co-inertia plots were done with Between Group Analysis using the Ade4 package in R (Dray and Dufour, 2007; R Core Team). Alpha and beta diversity metrics were calculated using Phyloseq version 1.13.4 (McMurdie and Holmes, 2013).

### Co-occurrence networks

The OTU table was filtered for OTUs with >10 reads in at least 5 of the 16 samples. A single co-occurrence network was built using relative abundances, and a positive correlation was defined as >98% Pearson's correlation and < 5% FDR (Benjamini–Hochberg adjusted; BH FDR). Sub-networks with more than 10 nodes were identified as major sub-networks, with all other nodes classified as not affiliated with a network. Large, unconnected sub-networks were identified and correlated to a sample site by percent abundance. Networks and sub-networks were created, examined, and visualized in R using the iGraph and ggplot2 packages (Csardi and Nepusz, 2006; R Core Team; Wickham, 2009).

### Metagenome binning and functional annotation

Metagenome assembly and binning was performed using MaxBin 2.0 (Huson et al., 2011; Wu et al., 2015). Briefly, short metagenomic reads from pond triplicates were combined and assembled using IDBA-UD (Peng et al., 2012). Short reads were then aligned to the co-assembled contigs using BWA to obtain the differential coverage of the contigs among each individual pond triplicate (Li and Durbin, 2009), which allows for tracking individual MAGs across multiple samples. The MaxBin 2.0 algorithm identifies the number of MAGs based on the presence of 40 marker genes that are shared among bacterial and archaeal genomes (Wu et al., 2013), and >500 bp contigs were binned by coverage and tetramer frequency. MAG completeness and contamination was determined with checkM (Parks et al., 2015), and quality reported according to the MIMAG standards (Bowers et al., 2017). Carbohydrate-active enzyme (CAZy) annotation was performed by identifying candidate open reading frames (ORFs) using Prodigal version 2.60, and searching with the dbCAN version 5.0 hidden markov models (Hyatt et al., 2010; Yin et al., 2012; Lombard et al., 2014). ORFs were additionally annotated for function using FOAM (Functional Ontology Assignments for Metagenomes) to obtain a list of KEGG Ontology numbers (Kanehisa et al., 2014; Prestat et al., 2014). FOAM annotation criteria including identifying the FOAM model with the highest HMM bit score >25. Differential abundance of FOAM abundances was done in R using DESeq2 1.14.1 using the Wald test, local fit type, and an Benjamini–Hochberg (BH) FDR of 5% (Love et al., 2014).

### Data availability

Metagenomic datasets are publicly available through the JGI IMG portal using the following IMG Taxon IDs: Pond 2C Water (3300000418, 3300000425, and 3300000369), Pond 2C Sediment (3300000409, 3300000892, and 3300000463), Pond 1C Water (3300000386, 3300000427, and 3300000371), Pond 1C Sediment (3300000515, 3300000374, and 3300000426), A23 Water (3300000381, 3300000526, and 3300000412) and A23 Sediment (3300000511, 3300001136, and 3300000488). MaxBin 2.0 MAGs (contigs and predicted protein sequences) are available at http://downloads.jbei.org/data/microbial_communities/microbial_communities.html.

## Results

### 16S rRNA gene sequencing

#### Quality control and filtering

Sequences from the V4 region of the 16S rRNA gene were clustered into 97% similarity OTUs, and further processed for quality and contamination removal. A total of 2,601 OTUs were identified among the 18 samples having at least 10 total reads. Thirty-nine of these OTUs were found to be phytoplankton chloroplast sequences comprising on average 19.7 and 1.7% of the water samples, and 1.1 and 0.24% of the sediments for Ponds 2C and 1C, respectively. A majority of these OTUs by abundance were assigned taxonomies of *Odontella* (84% of water and 74% of sediment chloroplast counts) followed by *Guillardia* (11% of water chloroplast counts, not identified in sediments). No OTUs of chloroplast origin were detected in the Pond A23 water or sediment samples. All chloroplast sequences were discarded from further analysis to focus on OTUs of bacterial and archaeal origin. Additionally, two of the replicates of the Pond A23 sediment samples had < 500 total reads and were discarded. In total, there were 2,562 OTUs in 16 samples.

#### Metrics of diversity

**Figure 1:**
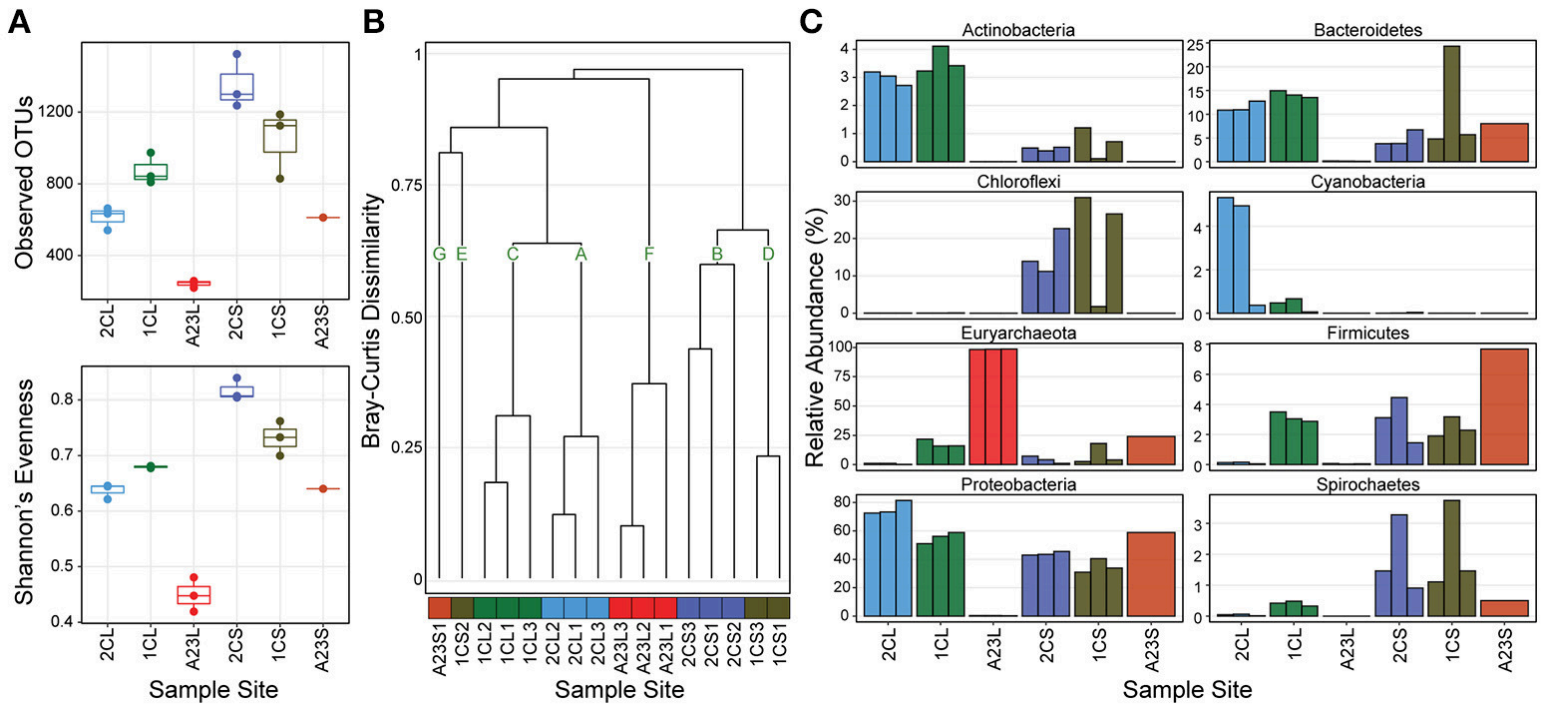
OTU abundance, diversity, and phyla of sampled ponds. Pond sample types are denoted with an “L” or “S” for water or sediment. **(A)** Richness and Evenness metrics for triplicates at each sample site (Pond A23 sediments has only a single replicate) **(B)** Bray-Curtis Dissimilarity dendrogram of all sample sites, color coded by sampled pond with assignment into seven groups at a cut-off of 0.6. **(C)** Relative abundance of the 8 most abundant phyla grouped by sample site.

We first determined the diversity of the samples, both within and across the ponds, and sample types. In the sediments, both the OTU richness and Shannon's evenness decreased with increasing salinity, however, in the water communities these metrics were highest in the 7.5% salinity Pond 1C, and lowest in the 33.2% salinity Pond A23 (Figure 1A). The diversity (both observed OTUs and evenness) was consistently higher in the sediments than for the water communities within all of the ponds. The low evenness of the Pond A23 water samples are due to two OTUs that combined are >60% relative abundance of the three replicates. Bray-Curtis Dissimilarity between sample sites revealed mostly similar community structures among replicates within a pond (Figure 1B). Using a Bray-Curtis similarity cutoff of 0.6, seven clusters (A–G) were identified. Samples types were more similar between Ponds 2C and 1C, with the water samples (clusters A and C) having highest similarity, and five of the six sediment samples clustered closely in B and D. The three Pond A23 water replicates were found in their own cluster F, and clusters G and E contain the remaining samples without similarity to other samples. Interestingly, although all of the Pond 1C water samples clustered together (cluster C), one of the sediment samples (1CS2) showed no strong similarity to any other sample, including its pair (1CL1) or the other Pond 1C sediments. This beta-diversity analysis revealed that the 2.5% and 7.5% salinity Ponds 2C and 1C had the highest diversity and were more similar to one another than Pond A23, revealing that the community structures are typically more similar among sample type (water or sediment) than by pond.

#### Community composition (16S rRNA gene sequences)

The abundance distribution of the most dominant phyla showed trends across the changes in salinity (Figure 1C). Euryarchaeota OTUs increased in relative abundance as salinity increased in both sample types, reaching >99% in the Pond A23 water samples but < 24% in the Pond A23 sediment sample. This steady increase of Euryarchaeota resulted in the decline of phyla dominant in the Pond 2C and 1C water samples, most notably the Proteobacteria, and Bacteroidetes. In the sediment samples, the Proteobacteria maintained their dominance in relative abundance, regardless of the Pond salinity. Further, even at the phylum level of resolution, the likely cause of the clustering differences (Figure 1B) between 1CS2 and its replicates can be seen to be due to an increase in Bacteroidetes with a large loss of Chloroflexi. Despite these few dissimilarities, overall Ponds 2C and 1C are highly similar, and the high-salinity Pond A23 has an abundance of halophilic Euryarchaeota.

Unclassified Bacterial OTUs were relatively abundant in Ponds 2C and 1C, ranging from 1.6 to 5.7% of water samples, and from 6.5 to 26.0% of the sediment sample abundances. Four of these (OTU_8, OTU_12, OTU_73, and OTU_100) were found at >1% relative abundance in at least one sample. OTU_12 was found in both the Pond 2C and 1C water samples and had a best hit of 93% to the Mollicutes HR1 genome (unpublished, NCBI BioProject PRJNA224116). The other three were found in both the Pond 2C and 1C sediments, all having a best match of only 84–85% to *Rubrobacter xylanophilus*, a thermophilic and radiation-resistant Actinobacteria (Ferreira et al., 1999). Additionally, there were few unclassified Archaea sequences, yet none of these were >1% relative abundance in any sample.

#### Sub-populations and microbial seed banks

**Figure 2:**
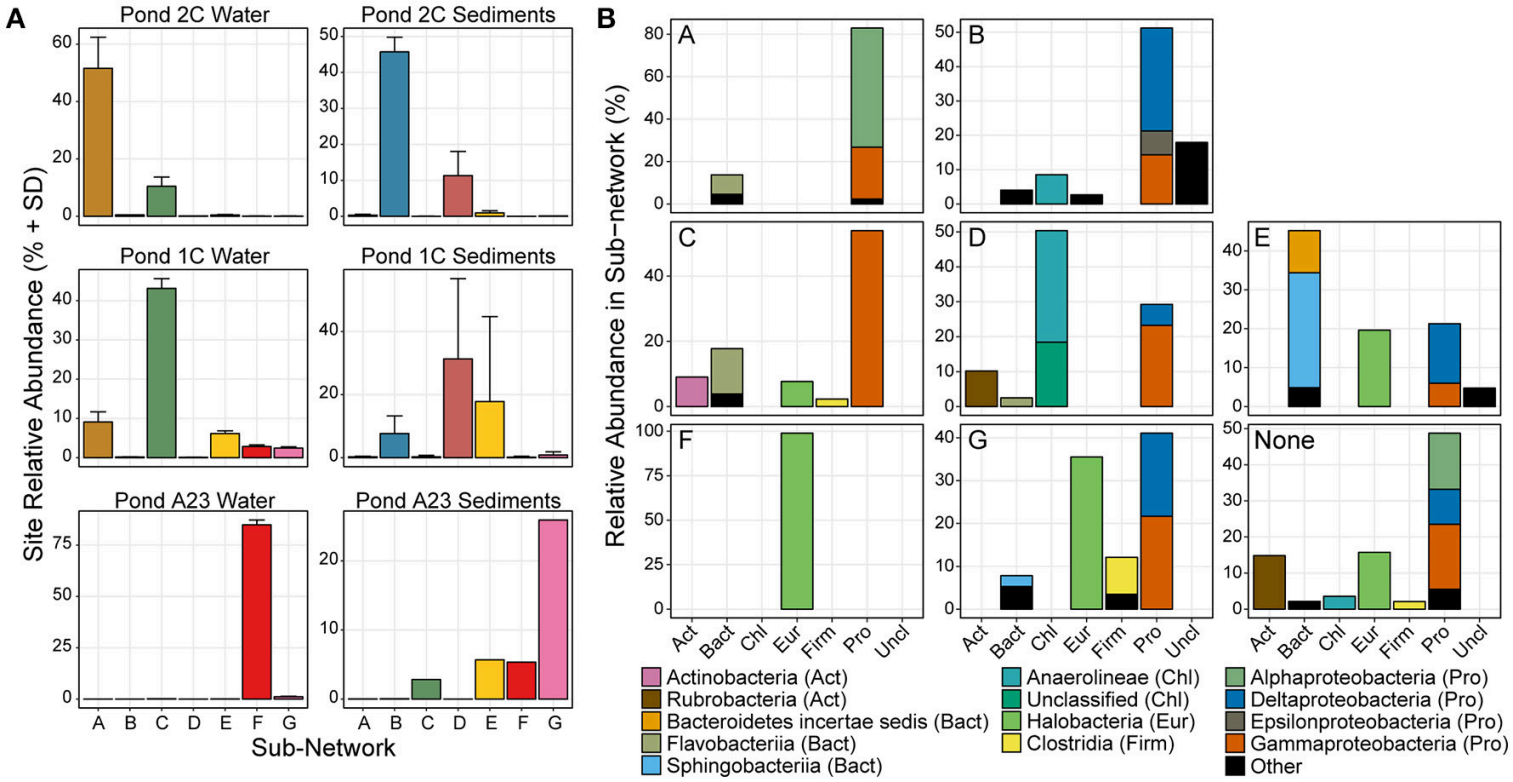
Relative abundance and phyla classification of seven main sub-networks. **(A)** The average relative abundance and standard deviation of OTUs in each of the 7 sub-networks show that certain microbial groups cluster strongly with a certain salinity or sample type. Only OTUs in the major sub-networks are shown. **(B)** Phylum distribution of OTUs in the 7 major sub-networks, colored by taxonomic class.

Given the proximity of the three ponds in this study, we were interested in the change in abundance of populations of species within the differing salinities and sample types. As water moves between ponds and undergoes salinity changes from rainfall, mixing with seawater, and/or evaporation, it carries microbial seed banks that are thought to awake from dormancy when conditions are ideal for that population (Lennon and Jones, 2011). We identified sub-populations (through co-occurrence networks of OTUs found in at least 5 sites, >98% Pearson's correlation, 5% Benjamini–Hochberg false discovery rate) of OTUs having similar relative abundance within the network regardless of pond or sample type. These sub-populations units, however, changed in relative abundance compared to other units among the different sample sites. Analysis of the distributions of these sub-networks across the different ponds allowed for defining groups of bacteria/archaea acting as microbial seed banks with low-abundance in certain environments, and higher abundance in others. Seven large sub-networks (>10 nodes) emerged from the 16S rRNA gene abundance data (Supplemental Figure 1 and Table 2), and a specific OTU could only belong to one of these seven sub-networks (A-G), or to none. Altogether, the OTUs in these seven sub-networks comprised 65% of the total read counts of all 16 samples. The sub-networks were largely partitioned according to pond and sample type (Figure 2A), which is expected given the vastly different conditions at each site (Berry and Widder, 2014). Our intent, however, is not to infer interactions between network members within a Pond [see (Jones et al., 2017)], rather our aim is to track groups of microbes that have a preference for a given salinity.

**Table 2:** Node and edge information about the major sub-networks from Figure 2.

**Sub-Network**	**Predominant Sample Sites**	**Observed OTUs (nodes)**	**Edges**	**Relative Read Abundance**
A	2C Water	82	400	11.5
B	2C Sediments	330	1,808	10.1
C	1C Water	111	358	10.3
D	1C 1 & 3 Sediments	134	635	8.1
E	1C 2 Sediments	99	836	5.1
F	A23 Water	52	298	16.8
G	A23 Sediments	69	515	2.5

Several trends were observed in the relative abundance of the seven sub-networks (A-G) across the range of salinities and sample types (Figure 2A). All sub-networks were found in multiple salinities and sample types, however sub-networks A, C, and F primarily defined the water samples, while B, D, E, and G were most dominant in the sediments. Overall, of the six sets of samples, there was a single network dominant in each set, with two conflicting sub-networks in the replicates of the mid salinity Pond 1C sediments. The dominant sample set for a given sub-network corresponds to the assignment of each sample to one of the seven previously identified clusters (Figure 1B), indicating the OTUs captured in these sub-networks largely drive the beta-diversity seen between the sampled ponds.

There were three primary sub-networks among the water columns samples; A, C, and F, and these were the dominant sub-networks for Ponds 2C, 1C, and A23, respectively. Sub-networks A and C are primarily Proteobacteria but are composed of different classes that shift between Ponds 2C and 1C (Figure 2B). Sub-network A is primarily Alphaproteobacteria, most of which are members of the Rhodobacteraceae family, while the Gammaproteobacteria OTUs are mostly Alteromonadaceae. Sub-network C, however, has lost all of the Alphaproteobacteria, replacing them with Gammaproteobacteria with sequence similarity to *Thiohalobacter*, a moderate halophile with a growth optimum of 0.5 M NaCl (Sorokin et al., 2010). Although both sub-networks A and C had a 15–20% Flavobacteriia component, these were primarily *Polaribacter* in sub-network A, and *Psychroflexus* in sub-network C, both members of the marine clade of Flavobacteriaceae (Bowman, 2006). Pond A23 was the most dissimilar to the water fractions from the other two ponds and was almost exclusively sub-network F and Halobacteria. More than 60% of the Pond A23 water samples were from only two OTUs (OTU_4 and OTU_1), both belonging to *Halonotius*. The third most abundant OTU in Pond A23/sub-network F belonged to *Haloquadratum*, which has been shown to have a lower abundance in winter as well as an inverse correlation with *Halonotius* (Podell et al., 2014). As our samples were collected in December, this could explain the relatively low abundance of *Haloquadratum* and the resulting dominance of *Halonotius* species.

The higher richness and complexity of the sediment samples resulted in more complex sub-networks, however, trends were still seen in response to the increasing salinity. Sub-network B was most abundant in the Pond 2C sediments, decreasing in abundance in the sediments as pond salinity increased. This sub-network consisted of a variety of taxa involved in sulfur cycling including both sulfate-reducers (of the Desulfobacteraceae family) and sulfur-oxidizers (of the Helicobacteraceae, Anaerolineaceae, and Ectothiorhodospiraceae families). These families of sulfate-reducers and sulfur-oxidizers have been found together in sediments of both cold (Eastern Mediterranean) and hydrothermal (Nankai Trough) marine methane seeps (Nunoura et al., 2012; Pop Ristova et al., 2015). There was one methanogenic archaeal OTU identified (>1% relative abundance) in this sub-network belonging to *Methanobrevibacter*, also found to be active in the Ninkai Trough (Newberry et al., 2004). Two different sub-networks (D and E) emerged as dominant within the Pond 1C sediment replicates, each largely displacing the other. OTUs of sub-network D were abundant in samples the 1CS1 and 1CS3 samples, while sub-network E was abundant in sample 1CS2 (Figure 1B). The D sub-network was characterized by an abundance of Chloroflexi (of the class Anaerolineae), while sub-network E had an abundance of Bacteroidetes (of the Sphingobacteriia class) as well as Halobacteria. These differences could reflect an unknown heterogeneity in the Pond 1C sediments that were not apparent visually such as a microbial mat. Sub-network E has many “salt-in” prokaryotic species including both Archaea as well as *Salinibacter*, while sub-network D is entirely “salt-out” organisms. Finally, the Pond A23 sediments were mostly sub-network G, which is a mixture of Delta- and Gammaproteobacteria, Halobacteria, and Clostridia OTUs. Within the Proteobacteria, the OTUs present belong to more halotolerant or halophilic taxa, including sulfate-reducing *Desulfohalobium*, “salt-out” *Halomonas*, and “salt-in” *Natronomonas*.

Although the sub-networks typically dominated a few specific sample sites, OTUs from all seven sub-networks were identified in all samples. This raises the possibility that these sub-networks represent collections of microbial taxa that remain in rare or low abundance in less favorable salinities yet are able to emerge as the dominant taxa in more favorable salinities.

### Metagenomics

#### Site comparison of FOAM gene abundances

**Figure 3:**
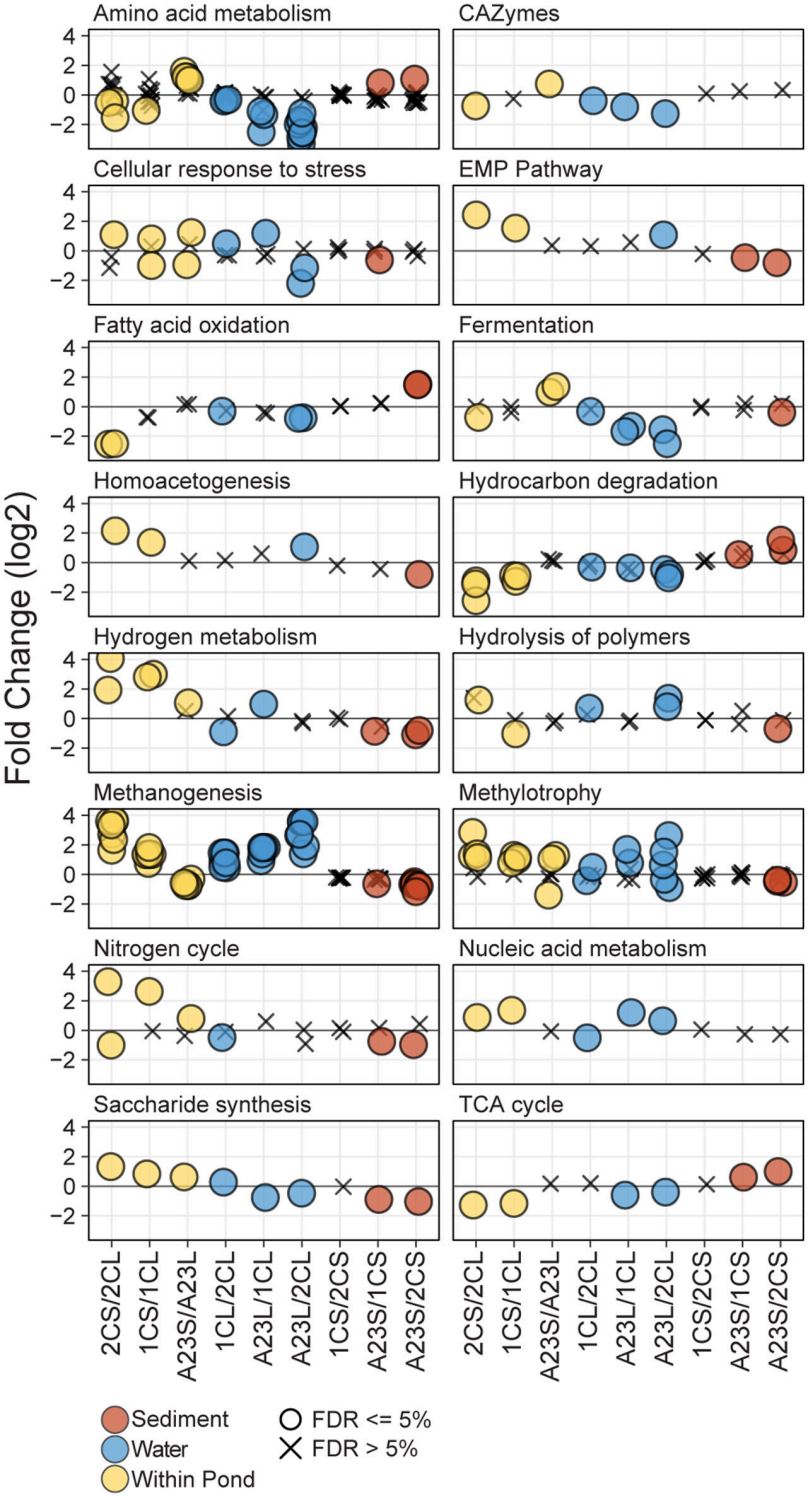
Differential abundance of FOAM L1 and L2 categories. Differential abundance of FOAM categories between different comparisons of the metagenomic data. For each comparison, negative numbers are higher in abundance for the sample listed first, and positive numbers are higher for the sample listed second. Points are represented as circles if significantly different (FDR ≤ 5%) and Xs if not significant. Each panel represents a FOAM L1 category, and each circle represents a FOAM L2 category. Comparisons include between water and sediment triplicates of a pond (yellow), between water samples from different ponds (blue) and between sediment samples from different ponds (brown). Comparisons are arranged from left to right with increasing salinity or increasing salinity differences.

To interrogate the differences in metabolic potential between the three ponds and fraction types, triplicate shotgun metagenomes from the water column and sediment fractions of each pond (18 total) were sequenced, assembled and annotated by the JGI. Predicted protein sequences were annotated using the Functional Ontology Assignments for Metagenomes database (FOAM; Prestat et al., 2014) and tested for significant differences (BH FDR = 0.05) in gene abundance within and between sample sites and fraction types. In total, there were 16 high level (L1) and 46 mid-level (L2) FOAM metabolism categories with significant differences among the comparisons revealing dissimilarities in functional potential with changes in salinity (Figure 3). Significant L1 and L2 differences were seen in all comparisons except for the Pond 2C and Pond 1C sediments, where no significant differences were observed.

Genes annotated with FOAM categories involved in osmotic stress showed various significant trends among and between ponds. In the water samples, there was a significant difference in abundance of cellular response to osmotic stress genes with a greater difference in salinity, with the higher salinity pond having more functional capability to cope with osmotic stress. The opposite was true for the sediment samples, with the high-salinity Pond A23 sediments having the generally having significantly less osmotic regulated genes than the lower salinity sediments. Within a pond, at lower salinities the sediments typically had a greater functional capacity to cope with osmotic stress, however, at the higher salinities the water samples typically had more osmotic stress-related genes than the sediments from the same pond. With the lower number of genes for osmotic stress response in the low salinity water samples came an increase in glycine betaine/proline transporters, while more glycine betaine/proline transporters were found in the high-salinity sediments compared to the high-salinity water samples. The only osmotic stress related transporter not correlated (positively or negatively) with salinity was for the general osmoprotectant ABC transporter of *opuA, opuBD*, and *opuC* (Kempf and Bremer, 1998) which was most abundant in the mid-salinity water and high-salinity sediment samples.

Amino acid metabolism gene abundance showed trends corresponding to salinity both within and between sample ponds. In the low salinity Pond 2C, there were more genes for glutamine/glutamate metabolism in the water column compared to the sediments. As salinity increased, however, this ratio changed with more in the high-salinity sediments than water samples. The same trend was observed for genes in histidine degradation. When comparing genes for these pathways among just the water column samples, the fold change was greater with increasing salinity difference between the compared samples, with Pond 2C having the most and Pond A23 having the fewest of these genes. Lysine catabolism was found to be a highly enriched pathway, particularly in the Pond 2C compared to Pond A23 water samples.

Genes involved in methanogenesis using seven different substrates (acetate, CO_2_, methylamine, dimethylamine, trimethylamine, formate, and methanol) displayed a strong salinity effect, both when comparing within and between ponds. Within a pond, the low-salinity Pond 2C sediments had more methanogenesis-related genes than the water column, and this ratio gradually inverted as salinity increased, with the Pond A23 water samples having more methanogenesis-related genes than the sediments. Comparisons between the pond water samples revealed more methanogenesis genes in the greater salinity pond, and the magnitude of the pathway enrichment increased with the salinity difference between the ponds. Between pond comparisons of the sediments showed less of a difference between salinities, however, it was opposite of the water samples in that the lesser salinity sediment generally had a greater abundance of methanogenesis genes.

#### Site comparison of CAZymes gene abundances and predicted substrates

**Figure 4:**
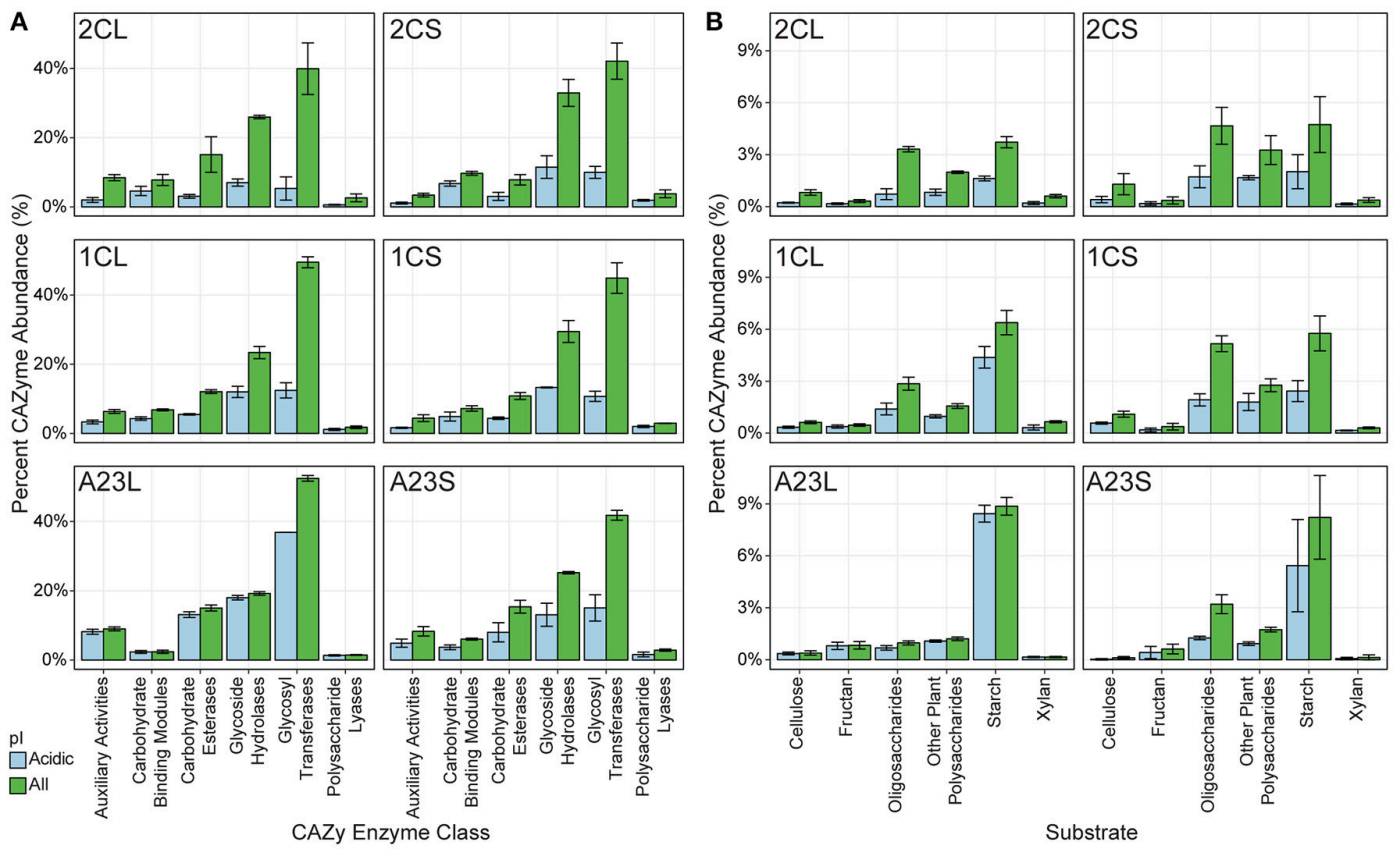
Predicted CAZy enzymes from the metagenomes. Predicted carbohydrate-active enzymes (CAZymes) were predicted for each pond, and each class was scaled to percent abundance relative to all cazymes. All genes are shown in green, and genes with a predicted isoelectric point less than or equal to 5.0 is shown in blue. Error bars represent the standard deviation among replicates for each pond and sample. **(A)** Distribution of CAZymes into CAZy class. **(B)** Distribution of GH CAZymes according to their predicted substrate.

We were particularly interested in mining the metagenomic data for novel salt-tolerant, carbohydrate-active enzymes (CAZymes) with potential applications in biofuel production (Lombard et al., 2014). Many CAZymes are exported, and therefore salt-tolerance is needed in high saline environments, regardless of the halotolerant strategy used by the organism (Oren et al., 2005). Fifty-one thousand six hundred and twenty putative CAZymes were identified from the entire metagenomic dataset, of which 19,277 had a calculated isoelectric point (pI) of 5.0 or less (Figure 4A). With an increase in salinity there was an increase in genes coding for CAZymes with a predicted pI below 5.0. On average, 27.0, 42.0, and 81.4% of the pond 2C, 1C and A23 water CAZymes are predicted to be acidic, compared to 36.0, 38.2, and 46.3% for the pond sediments.

In the pond sediments, only the auxiliary activity (AA), carbohydrate-binding module (CBM), and carbohydrate esterase (CE) CAZyme classes were significantly correlated (BH-corrected *p* ≤ 0.05) with changes in salinity, positively for AA and CE, and negatively for CBM. These significant differences in the sediments were lost at the finer resolution of individual CAZyme families, however, and only GT75, a glycosyl transferase (GT), and putative β-glucosyltransferase, was positively correlated with salinity. In the pond water samples, the CBM and glycoside hydrolase (GH) were both significantly negatively correlated with salinity. Individual CAZyme families, however, showed varied correlations with salinity. There were 26 different CAZymes with predicted GH activity, with GH15, GH77, and GH27 the most positively correlated with salinity, and GH23, GH74, and GH3 the most negatively correlated.

Of the AA class CAZymes, AA3, an enzyme family primarily found in fungi, was the most abundant in the Pond A23 water, while AA2 was the most abundant in the lower salinity ponds (Levasseur et al., 2013). CBM40 and CBM44 were the primary carbohydrate-binding modules with predicted affinities for sialic acid and cellulose, respectively (Moustafa et al., 2004; Najmudin et al., 2006). There was a significant shift in the GT gene families from low to high salinity, with GT41 and GT51 dominating lower salinities, and significantly more GT81 and GT66 glycosyl transferase genes at the high salinities. Although not significantly correlated with salinity, the archaeal GT2 and GT4 were prevalent at higher salinities, and are evolutionary widespread glycosyl transferases are involved in *N*-glycosylation (Magidovich and Eichler, 2009).

To explore the ecological context of the glycoside hydrolase (GH) enzymes, predicted GHs were grouped into functional families according to potential substrates including oligosaccharides, cellulose, chitin, plant polysaccharides, starch, fructans, xylans, or mixed substrates (Berlemont and Martiny, 2015). Overall, there were no significant shifts in substrate utilization in the pond sediments as a result of salinity changes (Figure 4B). In the water samples, however, several substrates had significant shifts with salinity, with starch and fructan utilization increasing with salinity, and cellulose, xylan, and various oligo/polysaccharide GH genes decreasing with increasing salinity.

Genes predicted to be involved with cellulose hydrolysis decreased in the pond water samples with increasing salinity. In bacteria, GH5 is the major cellulase (Berlemont and Martiny, 2013), and was the sole significantly different GH acting on cellulose, yet was on average only 0.5% of the CAZyme abundances. Other cellulases such as GH6, GH8, GH9, GH12, and GH44 were found sparingly and not correlated with salinity, and we found no evidence of GH45 or GH48 cellulases in any of the pond metagenomes, suggesting that cellulose is not utilized as a major nutritional component for these communities.

Most of the oligosaccharide-active GH genes identified in the metagenomes were β-glucosidases that hydrolyze cellobiose into glucose (GH1 and GH3), and GH2 β-galactosidases. GH3 genes were more abundant than GH2 in most ponds. The significant decrease in predicted oligosaccharide-degradation with increasing salinity was due to GH1, GH2, and GH3. Many of these genes did not have an acidic isoelectric point, with the exception of the GH3 CAZymes. Other dominant oligosaccharide-active GHs included α-glucosidase genes from the GH31 families that were found in similar relative abundances in all of the samples.

Genes for starch degradation were abundant in all of the pond metagenomes, particularly at higher salinities. While not significantly correlated with salinity, a majority of the putative starch-degrading genes were in the GH13 α-amylase. GH13 α-amylases are the major CAZyme with activity on α-glucosidic bonds, such as those found in amylose and starch (MacGregor et al., 2001). Given the high abundance of archaea, we also looked for GH57 type α-amylases, which are often found in thermophilic bacteria and archaea (MacGregor et al., 2001). A total of 250 GH57 genes were identified, however, over 75% of these were found in the low and mid-salinity sediments and were not correlated with the presence of haloarchaea or with salinity. The GH family driving the significant shift correlating starch utilization with salinity was GH15, including putative trehalases.

Despite the abundance of brine shrimp and the potential chitin carbon source (Sorokin et al., 2012) in the salt ponds, there were very few genes for chitin-utilization in the metagenomic data, and none correlated with salinity changes. All of the ponds had genes related to fructan degradation, namely GH32, however, the water sample from Pond A23 was the only sample with a prevalence of the putative levansucrase GH68 family. Halophilic bacteria such as *Halomonas* are able to produce industry-scale amounts of levan (Diken et al., 2015), and the Euryarchaeota may have evolved an abundance of GH68 genes to capitalize on this carbon source in the environment. Additional GH families detected were related to xylan and plant polysaccharide degradation, including GH16 and the endo-β-1,4-xylanase GH30, both negatively correlated with salinity.

#### Metagenome-assembled genomes

We reconstructed and functionally annotated 44 genomes from the nine water sample metagenomes that were >70% complete and < 10% contaminated (Parks et al., 2015). Forty-two MAGs could be placed within the Bacteria domain and two to Archaea, and were categorized into eight different phyla (Table 3). A majority of the MAGs belonged to either the Bacteroidetes (18 total) or Proteobacteria (16 total). The remaining MAGs were Actinobacteria (3 total), Cyanobacteria (2 total), Euryarchaeota (2 total), and one MAG each for Balneolaeota, Deinococcus-Thermus, and Verrucomicrobia. By relative abundance of the MAGs, the metagenomic data matched the 16S rRNA gene data that showed a predominance of Proteobacteria in Ponds 2C and 1C, and Euryarchaeota in the Pond A23 water samples.

**Table 3:** Metagenome assembled genome summary statistics.

**Bin**	**Pond**	**Mean abundance (%)**	**Phylum**	**Species**	**Size**	**GC%**	**Completeness**	**Contamination**	**N50/L50**	**MIMAG quality**
1CL.013	1C	1.49	Actinobacteria	*Candidatus Aquiluna* sp. IMCC13023	2.355 MB	47.6	84.09	7.14	275/1.87 KB	Medium
2CL.009	2C	2.46	Actinobacteria	*Candidatus Aquiluna* sp. IMCC13023	1.908 MB	46.3	81.58	3.95	290/1.426 KB	Medium
2CL.148	2C	0.13	Actinobacteria	*Actinobacterium* acMicro-4	1.289 MB	63.3	74.29	9.66	155/2.361 KB	Medium
1CL.008	1C	1.73	Bacteroidetes	*Salinibacter ruber*	3.280 MB	66.2	92.87	4.12	104/8.518 KB	High
1CL.019	1C	0.89	Bacteroidetes	*Salinibacter ruber*	3.738 MB	60.8	96.82	2.6	50/22.613 KB	High
2CL.082	2C	0.18	Bacteroidetes	*Salinibacter ruber*	4.236 MB	66.3	96.21	9.73	123/9.386 KB	Medium
A23L.016	A23	1.46	Bacteroidetes	*Salinibacter ruber*	3.320 MB	67.5	92.77	6.3	267/3.67 KB	Medium
2CL.078	2C	0.53	Bacteroidetes	*Flavobacteria bacterium* MS024-3C	3.029 MB	40.2	89.4	7.51	157/4.948 KB	Medium
2CL.132	2C	0.25	Bacteroidetes	*Flavobacteria bacterium* MS024-3C	3.071 MB	37.5	88.06	6.41	72/13.096 KB	Medium
1CL.009	1C	1.76	Bacteroidetes	*Flavobacteriales bacterium* BRH_c54	2.726 MB	39.2	93.84	5.59	101/7.134 KB	Medium
1CL.033	1C	0.69	Bacteroidetes	*Flavobacteriales bacterium* BRH_c54	2.976 MB	42.5	99.47	3.16	21/38.546 KB	High
2CL.020	2C	1.36	Bacteroidetes	*Flavobacteriales bacterium* BRH_c54	2.828 MB	39.3	92.25	6.94	98/7.709 KB	Medium
2CL.036	2C	0.63	Bacteroidetes	*Flavobacteriales bacterium* BRH_c54	3.056 MB	42.5	100	2.51	20/47.731 KB	High
1CL.017	1C	1.10	Bacteroidetes	*Fluviicola taffensis*	3.340 MB	39.6	98.12	3.72	152/4.233 KB	High
2CL.127	2C	0.12	Bacteroidetes	*Fluviicola taffensis*	3.760 MB	39.8	90.54	4.11	148/7.189 KB	High
1CL.124	1C	0.16	Bacteroidetes	*Nonlabens ulvanivorans*	2.013 MB	36.6	80.09	9.44	202/3.024 KB	Medium
1CL.030	1C	0.59	Bacteroidetes	*Owenweeksia hongkongensis*	2.773 MB	54.4	93.9	3.88	161/4.92 KB	High
1CL.011	1C	1.34	Bacteroidetes	*Psychroflexus tropicus*	1.809 MB	40.5	81.16	3.95	112/4.991 KB	Medium
1CL.025	1C	0.61	Bacteroidetes	*Psychroflexus tropicus*	2.867 MB	32.5	72.57	1.72	289/2.645 KB	Medium
1CL.034	1C	0.57	Bacteroidetes	*Psychroflexus tropicus*	3.021 MB	36.5	72.93	9.71	100/7.521 KB	Medium
2CL.086	2C	0.34	Bacteroidetes	*Psychroflexus tropicus*	2.131 MB	40.2	89.27	4.36	72/8.867 KB	Medium
1CL.054	1C	0.26	Balneolaeota	*Gracilimonas tropica*	2.521 MB	51.2	94.53	5.76	146/5.237 KB	Medium
1CL.112	1C	0.20	Cyanobacteria	*Geitlerinema* sp. PCC 7105	3.831 MB	51.7	89.93	5.83	356/3.415 KB	Medium
2CL.007	2C	1.60	Cyanobacteria	*Geitlerinema* sp. PCC 7105	4.253 MB	51.4	95.08	3.06	145/8.176 KB	High
2CL.108	2C	0.23	Deinococcus-Thermus	*Truepera radiovictrix*	2.092 MB	74.3	82.19	3.8	172/3.559 KB	Medium
A23L.001	A23	12.05	Euryarchaeota	*Halophilic archaeon* J07HB67	2.854 MB	67.4	98.44	4.8	7/157.429 KB	High
A23L.007	A23	6.55	Euryarchaeota	*Haloquadratum walsbyi*	3.747 MB	47.5	89.79	4.32	352/2.847 KB	Medium
1CL.066	1C	0.31	Proteobacteria	*Octadecabacter antarcticus*	3.826 MB	65.9	88.18	8.55	176/5.738 KB	Medium
2CL.010	2C	1.83	Proteobacteria	*Roseobacter* sp. AzwK-3b	2.959 MB	62.7	88.2	7.71	208/4.355 KB	Medium
2CL.040	2C	0.65	Proteobacteria	*Beta proteobacterium* KB13	2.281 MB	34.3	71.49	5.99	112/4.087 KB	Medium
2CL.126	2C	0.17	Proteobacteria	*Escherichia coli*	1.766 MB	46.1	86.32	2.85	67/6.491 KB	Medium
2CL.073	2C	0.40	Proteobacteria	*Gamma proteobacterium* HIMB30	1.620 MB	55.6	72.76	0.8	9/52.317 KB	Medium
1CL.100	1C	0.17	Proteobacteria	*Glaciecola* sp. HTCC2999	1.200 MB	46.7	75.69	7.36	116/3.265 KB	Medium
2CL.008	2C	2.97	Proteobacteria	*Glaciecola* sp. HTCC2999	2.341 MB	40.2	85.04	4.68	150/3.985 KB	Medium
2CL.059	2C	0.63	Proteobacteria	*Glaciecola* sp. HTCC2999	2.673 MB	41.0	93.51	5.42	74/9.918 KB	Medium
2CL.052	2C	0.41	Proteobacteria	*Luminiphilus syltensis*	3.632 MB	59.6	98.19	4.19	14/73.751 KB	High
2CL.181	2C	0.11	Proteobacteria	Marine *gamma proteobacterium* HTCC2080	3.207 MB	56.9	70.35	8.82	431/2.071 KB	Medium
1CL.056	1C	0.33	Proteobacteria	*Marinobacter* sp. HL-58	3.337 MB	57.8	97.64	4.36	99/9.193 KB	High
2CL.120	2C	0.27	Proteobacteria	*Marinobacterium* sp. AK27	2.950 MB	48.2	94.43	3.41	112/6.605 KB	High
1CL.020	1C	0.74	Proteobacteria	*Pantoea ananatis*	0.993 MB	38.6	76.39	3.7	14/17.305 KB	Medium
1CL.001	1C	17.14	Proteobacteria	*Spiribacter* sp. UAH-SP71	1.456 MB	65.5	79.85	4.24	99/4.366 KB	Medium
1CL.036	1C	0.69	Proteobacteria	*Spiribacter* sp. UAH-SP71	2.845 MB	65.2	94.56	4.54	53/13.54 KB	High
2CL.002	2C	6.34	Proteobacteria	*Spiribacter* sp. UAH-SP71	1.694 MB	65.5	86.91	5.66	92/5.198 KB	Medium
2CL.003	2C	5.54	Verrucomicrobia	*Coraliomargarita akajimensis*	1.819 MB	68.7	72.64	6.03	308/1.656 KB	Medium

*Pond indicates the samples the genome bin was reconstructed from. Taxonomy, completeness, and contamination were predicted by CheckM*.

**Figure 5:**
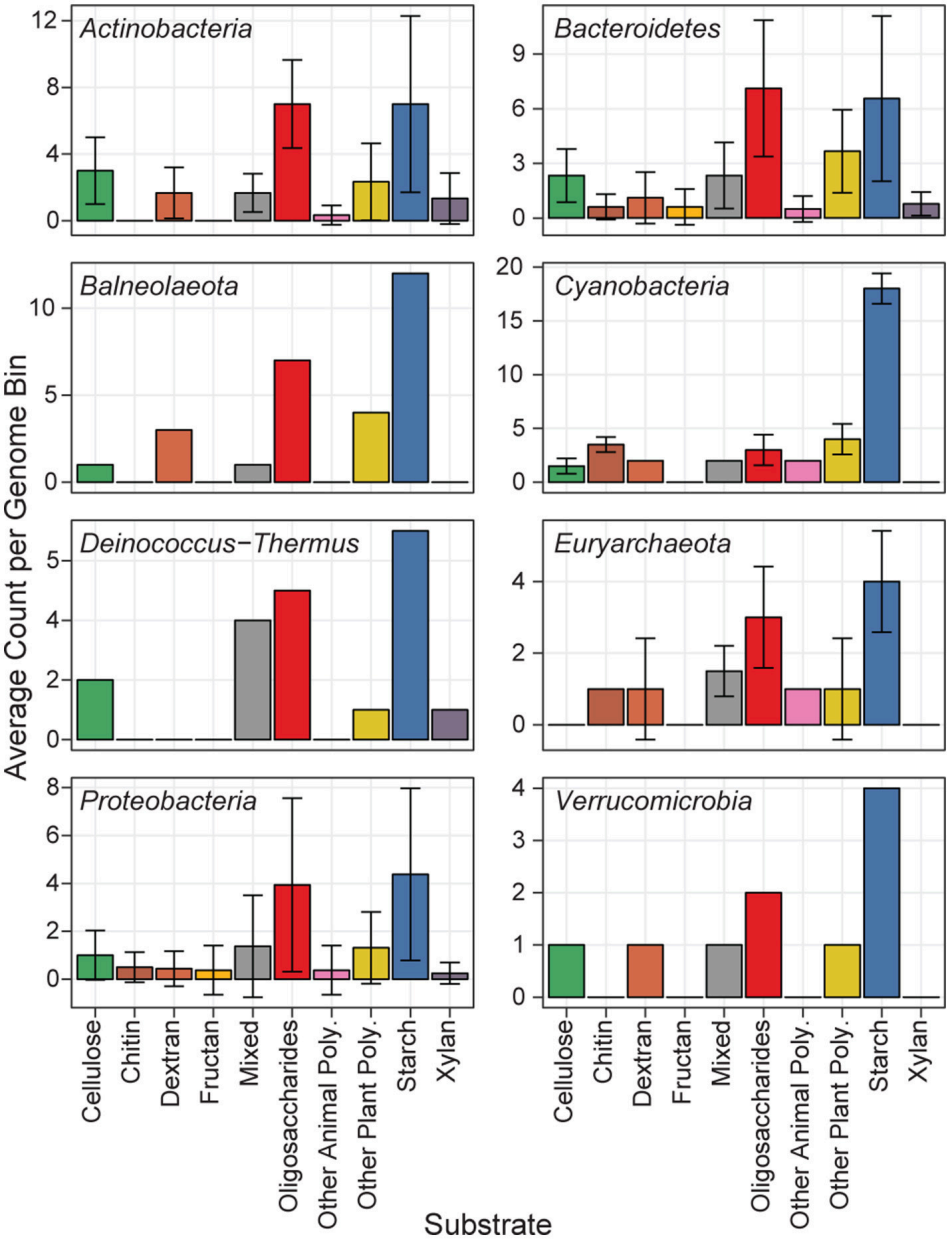
Distribution of MAG CAZymes by substrate. Each of the 44 MAGs is represented in one of eight phyla. The mean abundance of GH CAZymes according to their predicted substrate is shown, and error bars show the standard deviation.

Predicted proteins from the MAGs were annotated for carbohydrate active enzymes, and grouped according to their predicted substrate (Figure 5). All phyla showed functional capability to degrade a range of carbohydrates, particularly starch, and oligosaccharides. The Actinobacteria MAGs had the most cellulase genes per genome, and are likely cellulose degraders (Berlemont and Martiny, 2013), while the Euryarchaeota MAGs had no predicted cellulases. The newly classified Balneolaeota phylum (formerly a Bacteroidetes) and Cyanobacteria MAGs each had a relatively high amount of CAZymes with starch as a predicted substrate (Hahnke et al., 2016). Cyanobacteria have previously been shown to store glucose photosynthate as α- and β-1,4 linked polysaccharides which can later be hydrolyzed as a glucose source (Stuart et al., 2015). The two Cyanobacteria and Balneolaeota MAGs each had over 10 predicted GH13 genes, and the Cyanobacteria MAgs additionally each contained five GH57 genes. GH13 and GH57 are α-amylase enzymes acting on α-1,4 linkages, and could be used to hydrolyze the Cyanobacterial storage polysaccharides.

## Discussion

Salinity remains one of the strongest influences on microbial community composition, and several recent studies have been undertaken at a variety of locations aiming to better understand this diversity (Lozupone and Knight, 2007; Ghai et al., 2011; Fernández et al., 2014; Podell et al., 2014; Simachew et al., 2015; Ventosa et al., 2015). Here we characterized the microbial community composition as well as the metabolic and polysaccharide-degrading potential of three former saltern ponds with salinities of 2.5, 7.5, and 33.2%.

Co-occurrence network analysis of 16S rRNA gene abundances have been used to survey and infer bacterial interactions in complex environments (Barberán et al., 2012; Faust and Raes, 2012; Williams et al., 2014). When clustering samples from widely different environments, however, the networks that emerge are likely due to habitat preference, rather than actual interactions or dependence between co-occurring taxa (Berry and Widder, 2014). We used this feature of networks not to infer relationships, but to define core groups of microbes that can define or indicate a specific pond type, acting as a microbial seed bank. The emergent clusters of microbes co-occur in similar proportions throughout all of the sample sites they are found in, with only slight shifts in relative proportions. Additionally, these seven sub-networks grouped the ponds into the same groupings as the Bray-Curtis clustering at 0.6 dissimilarity (Figure 1B).

By defining a sample's core taxa as clusters of co-occurring organisms, tracking the abundance of these core groups of organisms across spatial or temporal distances becomes feasible. For example, networks “A” and “C” have opposing abundance patterns in the Pond 2C and 1C water samples, and while a low salinity pond can enrich for the “A” network cohort, the “C” network cohort remains largely intact, albeit at a low abundance. With the core microbial clusters for a range of salinities being present within a pond, the microbial populations can remain resilient and adapt to even radical changes in salinity. A moderate salinity of 15% (~2.5 M NaCl) is generally regarded as the transitional salinity for survival of marine microbes and moderate halophiles compared to extreme halophiles (Andrei et al., 2012). One possible explanation for the alpha-diversity peak that we observed in the mid-salinity Pond 1C is that as the 2.5 M salinity level was approached, the selection pressure was not yet too high to exclude the halotolerant and moderate halophiles, yet sufficiently saline for extreme halophiles to begin to increase in abundance. The 7.5% salinity Pond 1C contained between 15 and 20% extremely halophilic archaeal 16S sequences in the water samples, and between 3 and 18% in the sediments, contrasting with previous studies that found low abundance of these archaea in saltern ponds with < 11% salinity (Benlloch et al., 2002).

New metagenome binning approaches allow for the reconstruction of microbial genomes using nucleotide composition and differential coverage among multiple samples (Albertsen et al., 2013; Wu et al., 2015). From the water metagenomes we reconstructed and functionally annotated 42 bacterial and 2 archaeal MAGs. We mined the reconstructed MAGs with the aim of providing a resource for biofuel applications, particularly in the deconstruction of lignocellulolytic biomass. Previous studies have examined the role of certain phyla as opportunists in utilizing cellobiose/cellotriose that other organisms have released from larger polysaccharides (Berlemont and Martiny, 2013). Indeed, from the MAGs we have identified many of these potential “cheaters” among the Proteobacteria and Bacteroidetes. Most genomes belonging to these phyla have genes encoding β-glucosidases for disaccharide hydrolysis, yet they lack an endocellulase or other oligosaccharide-degrading enzyme. However, given that most MAGs appeared to lack functional cellobiose/cellotriose uptake mechanisms, it remains unclear how these substrates are being imported. We note that we cannot exclude the possibility that, due to the draft nature of the MAGs, genes for these uptake mechanisms may be present yet not binned with the majority of the genome.

We found a high abundance of GH15 trehalase genes in the metagenomes belonging to Euryarchaeota. Trehalose is a common osmolyte in “salt-out” microbes, and can be metabolized into two glucose molecules by GH15 enzymes (Sakaguchi et al., 2015). The high-abundance of GH15 enzymes in “salt-in” archaea could be a result of their utilization of bacterial-produced trehalose from “salt-out” organisms. Production of trehalose is widespread in certain lineages of Halobacteria (Youssef et al., 2014), and an additional role for GH15 trehalases could be to provide a means for recycling trehalose when no longer needed for osmoadaptation.

The lower salinity ponds were enriched for lysine degradation genes. Degradation of lysine could be beneficial in that it can produce acetyl-CoA for the citric acid cycle, however, there is also a role for lysine degradation products in relieving osmotic stress. Lysine degradation through the lysine dehydrogenase route was found to be upregulated in high-salt conditions, linking lysine metabolism to osmotic stress responses (Neshich et al., 2013). In addition to acetyl-CoA, proline and pipecolate are produced as degradation products, potentially acting as osmolytes to alleviate osmotic stress (Neshich et al., 2013).

Starch degradation genes are one of the most abundant classes of enzymes in each environment and MAG in this study. Phototrophic Cyanobacteria have been shown to convert photosynthetic product into external polysaccharide storage molecules during high-light, followed by a shift to a heterotrophic lifestyle under low-light conditions and utilizing these carbon stores (Stuart et al., 2016). As such, our Cyanobacterial MAGs show little capacity to degrade complex polysaccharides but have an abundance of α-amylase enzymes for mobilizing stored carbon (Stuart et al., 2016). We hypothesize that other microbes in the community evolved to utilize this photosynthetic product as a carbon source.

Hypersaline environments have previously been proposed as an ideal study site for the degradation of aromatic and phenolic compounds, including terrestrial runoff of lignin and its derivatives (Le Borgne et al., 2008; Fathepure, 2014). An acidic isoelectric point (pI) is a widespread feature of the proteomes of many halophilic archaea and a few halophilic bacteria (Oren, 2013) and these microbes, or the enzymes they produce, may be well-suited for industrial processes (van den Burg, 2003; Delgado-García et al., 2012). For example, ionic liquids (ILs) have tremendous potential for the pretreatment of plant biomass over other methods, a necessary step for conversion of cellulose into glucose for next generation biofuels (Li et al., 2010). ILs, however, can be extremely toxic to microbes and enzymes, and IL removal comes at a great expense. Halotolerant microbes or their enzymes have shown great potential to withstand IL concentrations far greater than typical lab strains, and are a potential resource for engineering IL-tolerance in biofuel-producing lab strains (Pottkämper et al., 2009; Gunny et al., 2014; Portillo and Saadeddin, 2014; O'Dell et al., 2015). Here we found that Pond A23 had a ten-fold increase in dissolved organic material over that found in Pond 2C (Table 1), with a corresponding abundance of CAZy AA genes primarily in the Proteobacteria and Bacteroidetes, as well as genes for low-pI AA proteins in the Euryarchaeota. These acidic lignin-degrading enzymes can be explored for their potential in converting the lignin in the waste-stream from ionic liquid pretreated biomass to high-value end products. In addition, these enzymes may prove useful in microbial remediation of aromatic and phenolic contaminants in natural saline environments.

Here we successfully constructed 44 of bacterial and archaeal genomes, including many novel taxa. These include genomes of some marine Alphaproteobacteria such as *Marinobacter* and *Roseobacter* that associate with phytoplankton, and *Roseobacter* sp. even have demonstrated lignin-deconstruction capabilities (González et al., 1996; Geng and Belas, 2010). Several genomes of *Glaciecola*, members of the Gammaproteobacteria, were also binned and some species in this genera have also demonstrated ability to depolymerize both cellulose and xylan (Guo et al., 2009; Klippel et al., 2011; Qin et al., 2014). In addition to genomes with similarities to known isolates, we binned many genomes from previously undescribed taxa without sequenced representatives (Table 3).

In summary, this study demonstrates that high saline environments such as saltern ponds are a tremendous resource for mining novel microbes and enzymes with potential applications for processing next-generation biofuels, particularly under high-salt conditions.

## Author contributions

JK, NB, TH, BS, SS, and JJ designed the experiments. JK, NB, Y-WW, and MD collected and analyzed experimental data. JK and JJ wrote the manuscript.

### Conflict of interest statement

The authors declare that the research was conducted in the absence of any commercial or financial relationships that could be construed as a potential conflict of interest.

## Supplementary material

Supplemental Figure 1 Co-occurrence network. Network of the 7 largest clusters, outlined by cluster number (see legend in B). Nodes are colored by phylum and sized according to total OTU abundance. Edges represent Pearsons correlation score of 0.98 or higher.
