# Supplementary figures and images for: Microbial Community Structure and Functional Potential Along a Hypersaline Gradient
Source: Front Microbiol. 2018 Jul 10;9:1492. doi: 10.3389/fmicb.2018.01492 (PMC6048260; doi:10.3389/fmicb.2018.01492)

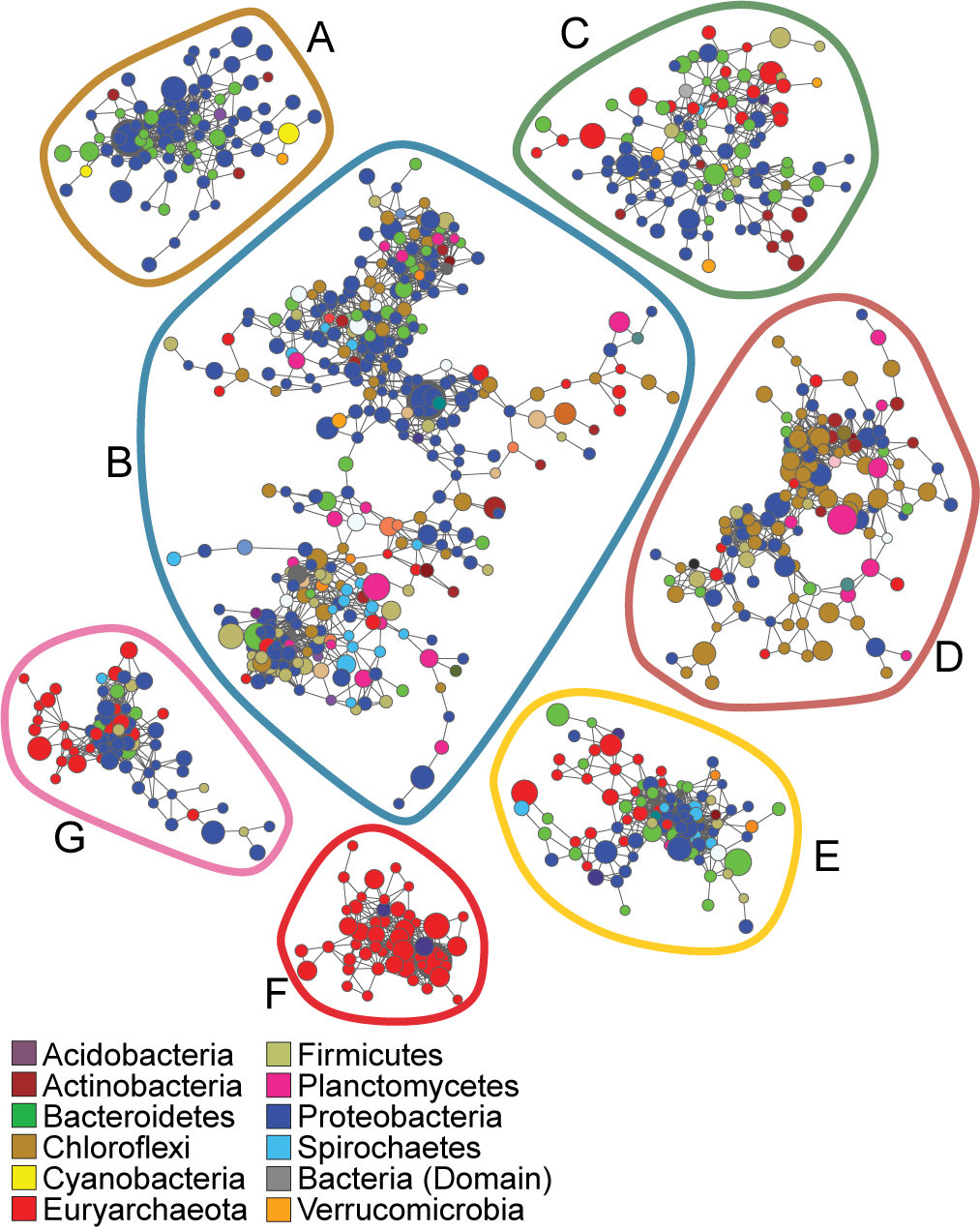

Supplement: Supplemental Figure 1 — Co-occurrence network. Network of the 7 largest clusters, outlined by cluster number (see legend in B). Nodes are colored by phylum and sized according to total OTU abundance. Edges represent Pearsons correlation score of 0.98 or higher. [file Image_1.TIF]
